# COVID-19: Knowns, Unknowns, and Questions

**DOI:** 10.1128/mSphere.00203-20

**Published:** 2020-03-18

**Authors:** Stuart Weston, Matthew B. Frieman

**Affiliations:** a Department of Microbiology and Immunology, University of Maryland School of Medicine, Baltimore, Maryland, USA

**Keywords:** COVID-19, SARS, coronavirus, SARS-CoV-2

## Abstract

The recent emergence of severe acute respiratory syndrome coronavirus 2 (SARS-CoV-2) from the Hubei province in China in late 2019 demonstrates the epidemic potential of coronaviruses. The rapid spread of this virus across the world in only 2 months highlights the transmissibility of this family of viruses and the significant morbidity and mortality that they can cause. We highlight the current state of knowledge of coronavirus biology while answering questions concerning the current outbreak of SARS-CoV-2.

## COMMENTARY

Until the very end of 2019, there were six coronaviruses known to cause disease in humans. Four of these result in little more than a common cold and are endemic around the world. The viruses known as human coronavirus (hCoV)-229E, hCoV-HKU1, hCoV-NL63, and hCoV-OC43 are of little concern at a global public health level. The other two, however, have caused more widespread concern. In 2002, severe acute respiratory syndrome coronavirus (SARS-CoV) emerged in the human population. In a matter of months, this virus from a bat that transmitted via a palm civet to infect a human in the Guangdong province of China infected over 8,000 people, killing roughly 10% (1). In 2003, SARS-CoV infections stopped, and the virus has not been seen since. A second epidemic coronavirus, known as Middle East respiratory syndrome coronavirus (MERS-CoV), emerged in 2012. Like the SARS-CoV outbreak, MERS-CoV started with a patient suffering pneumonia and came from a zoonotic event (this time from a bat via a camel to a human) (1). However, MERS-CoV has shown far more limited human-to-human transmission than SARS-CoV. Since 2012, there have been roughly 2,500 cases of MERS-CoV, mostly confined to regions of the Middle East. While case numbers are low for MERS-CoV, there is a high case fatality ratio (CFR) of approximately 35%, making this virus one of the deadliest human pathogens. Coronaviruses that infect humans all appear to have respiratory transmission, making them pathogens of pandemic potential. The end of 2019 saw the emergence of a novel human coronavirus that is rapidly spreading around the global and has a higher degree of lethality than the endemic coronaviruses, though not to the level of SARS-CoV or MERS-CoV. The virus was initially named 2019-nCoV but is now termed SARS-CoV-2 and causes the disease COVID-19 (coronavirus disease 2019). At the time of writing, there have been over 115,000 cases and over 4,000 deaths.

The first case of COVID-19 was reported to the WHO by Chinese authorities on 31 December 2019 as a result of a patient suffering pneumonia in Wuhan City, Hubei Province, China. Over the following days, more patients were suspected to be suffering the same disease, and by 9 January, a novel coronavirus had been detected and the sequence was published online shortly thereafter (2). The 2 months since emergence of SARS-CoV-2 have demonstrated the rapid pace at which a virus can spread and which science can develop. After an initial lag phase, cases of COVID-19 followed a closely exponential curve. The vast majority of cases are, at the time of writing, still from mainland China. However, over 100 other countries have reported cases. Most cases outside China have been associated with travel to that country, but more clusters of cases are now being detected without travel history. In this article, we will discuss what has already been learned about the virus and will then outline 10 key questions and directions of study for this novel coronavirus.

## 

### SARS-CoV-2 virology.

The novel virus is in the *Coronaviridae* family and therefore shares common features of this family. Coronaviruses have large (∼30-kb) single-stranded, positive-sense RNA genomes. The genome can be roughly divided into a 5′ two-thirds and a 3′ third. The first two-thirds of the genome code for two large polyproteins (pp1a and pp1ab from ORF1a and ORF1b) which are proteolytically cleaved into the nonstructural proteins (nsp1 to -16) that are essential for production of new viral genetic material. The remainder of the genome codes for the structural proteins and carries the accessory genes that produce virions and alter the host response, respectively (1).

As the naming may suggest, the newly emerged coronavirus is closely related to SARS-CoV, sharing roughly 80% identity at a nucleotide level. The closest relative of SARS-CoV-2 appears to be a virus found in bats known as RaTG13-2013 (96% identity), suggesting that, similarly to SARS-CoV, the virus entered the human population from a spillover event either directly from a bat or through an animal intermediate (3). Early studies on SARS-CoV-2 have shown further similarities with its namesake virus in that the spike protein utilizes ACE2 as its cell surface receptor (3, 4). ACE2 is found on ciliated epithelial cells of the human lungs, and this receptor utilization influences the tropism of these viruses.

Based on the speed at which the outbreak of COVID-19 has developed, SARS-CoV-2 appears to spread easily in the human population. Many health care workers have been infected, and more clusters of cases are being detected with each passing day. The reproductive number (*R*_0_) of the virus is currently thought to be around 3 (5), again suggesting the potential for sustained human-to-human transmission that appears to be through respiratory droplets and potentially a fecal-oral route (6).

While much can be gleaned from our general understanding of coronavirus biology and some of the early studies on SARS-CoV-2, many pertinent questions exist, for which we will outline 10 here.

### (i) What is the animal reservoir/intermediate host?

The bat virus termed RaTG13-2013 has 96% identity with SARS-CoV-2 (3), strongly pointing toward a shared common ancestor and suggesting that the novel human pathogen originated in bats. When the zoonoses first occurred remains an interesting question. Both SARS-CoV and MERS-CoV also have common ancestry with viruses found in bats. Both of these viruses had an intermediate host for transmission into humans, these being palm civets and camels for SARS- and MERS-CoV, respectively (1). There has been a suggestion that pangolins may be the intermediate host for SARS-CoV-2 (7), but this remains to be established. Knowing the intermediate host is an important step for understanding how SARS-CoV-2 became a human virus and how to potentially curtail further spillover events. Knowing that MERS-CoV transmits to humans via dromedary camels has allowed for the development of a camel vaccine to potentially limit spread to humans (8, 9). Approval for novel vaccines in animal hosts is far easier than in humans. Moreover, knowing the intermediate host allows for measures to be taken to limit human contact with the animal (e.g., not selling meat from these animals in wet food markets), which can again help reduce the chances of future spillover events. However, with the spread of SARS-CoV-2 being so extensive, whether these measures would be effective at limiting human cases of COVID-19 may be unlikely.

### (ii) What is the true case count (mild symptoms/asymptomatic carriers) and lethality?

Since the early stages of the COVID-19 outbreak, the case fatality ratio (CFR) has been around 2% to 4%. This is a much lower CFR than those seen for SARS and MERS, which are around 10% and 35%, respectively. However, many people remain in severe conditions in hospitals as a result of COVID-19, which could see the CFR increase. With that said, the true burden of disease in the human population is currently unknown. A recent report on approximately 44,000 Chinese patients showed that 81% develop only mild symptoms, while 14% develop severe symptoms and 5% become critically ill (10). This study suggests that even among the people with symptoms severe enough to be tested for SARS-CoV-2, there can still be mild symptoms, which may suggest that many other people have symptoms mild enough to not require testing. This would make the true case count far larger and, by extension, the CFR lower. Early estimates suggested that the true case count may be as much as 10 times higher than was being reported (11). Establishing the true disease burden in the human population will take time and retrospective serological survey and is a secondary aspect in relation to controlling the current disease outbreak. But it remains an interesting question to be determined moving forward.

### (iii) What comorbidities are associated with severe disease outcome and how do these affect viral pathogenesis?

As more cases of COVID-19 occur, it is becoming established that the most severe cases and mortality are associated with underlying health conditions. The most common associated comorbidities are pulmonary disease, diabetes, and old age (10). Interesting questions as to how these comorbidities impact viral pathogenesis are open for investigation. More severe SARS cases were also associated with age, and work in mice has demonstrated this (12–14). Severe MERS is associated with diabetes and other underlying health conditions (15, 16), and again, work in mice has shown that diabetes can impact the immune response to infection, leading to increased pathogenesis (17). It will be interesting to see whether SARS-CoV-2 infection is similarly impacted.

### (iv) Can spread of SARS-CoV-2 be contained/will the virus persist in the human population?

Four of the seven human coronaviruses are endemic around the world but cause little more than the common cold. Currently, SARS-CoV-2 is a global epidemic, with the potential to be considered a pandemic. In one scenario, this outbreak may be contained, and the virus never seen again, like SARS-CoV. Alternatively, the virus may become an endemic virus with seasonality like influenza and the other human coronaviruses. However, it is too early to know whether SARS-CoV-2 spread will be affected by changing weather conditions. Nearly all cases of COVID-19 have been in China, where it is winter; whether cases will decrease as temperatures increase in the Northern Hemisphere, as is seen for influenza, remains to be seen.

### (v) What *in vitro* and *in vivo* systems can be used for research?

A very important question for understanding SARS-CoV-2 infection is what systems can be used for study. Early studies on SARS-CoV-2 determined that the cellular receptor for the virus is ACE2, similarly to SARS-CoV (3). This knowledge helps to develop an understanding of susceptibility of certain *in vitro* cell lines to infection with the novel virus. The likelihood is that if cells were not permissive for growth of SARS-CoV, they probably will not support growth of SARS-CoV-2. As more labs around the world start researching the new virus, a better understanding of the permissive cell lines will be developed, an important step to testing therapeutic options and developing a better understanding of basic aspects of SARS-CoV-2 virology. The more challenging aspect of lab-based research on the novel human coronavirus will be developing small-animal models. The early research on receptor usage suggests the virus is not able to infect cells expressing mouse ACE2 (3), thus making a mouse model potentially challenging. Whether expression of human ACE2 in mouse lungs using adenovirus or mouse adaptation of SARS-CoV-2 can develop appropriate models, as was done for SARS-CoV (18), is a pressing question. Whether other small-animal models can be used also needs to be investigated. These models will be essential for thoroughly testing therapeutic candidates and vaccine strategies and understanding the pathology of disease.

### (vi) Can we find therapeutic options?

As the case count and death toll of the epidemic continue to increase, it becomes imperative to identify therapeutic options for COVID-19. Once *in vitro* and *in vivo* systems have been established, these tests can proceed. Drug repurposing may prove to be the best strategy for quick development of novel therapeutic options. A novel therapeutic being tested is remdesivir (19, 20), which in combination with chloroquine has been found to inhibit SARS-CoV-2 growth *in vitro* (21). It was recently announced by the NIH that remdesivir would be entering phase 3 clinical trials in humans. Chloroquine has also been reported to be effective in patients in China (22). A combination of lopinavir and ritonavir is also under investigation in human cases of COVID-19 in China. Many more people will need to be treated with these drugs to determine true efficacy, but they are promising leads.

### (vii) Can a vaccination strategy be developed?

An effective strategy to contain virus spread is vaccination. During the SARS-CoV and MERS-CoV outbreaks, much research went into developing vaccine strategies (23). However, the cessation of the SARS epidemic and the minimal human-to-human transmission of MERS-CoV have curtailed the testing of these interventions in humans. With the emergence of SARS-CoV-2, a new impetus into development of coronavirus vaccines has been generated. There are several platforms being used to develop vaccines against SARS-CoV-2, including spike subunit, DNA, RNA, whole-virion, and nanoparticle vaccines. Future testing in cells and animal models will determine which is most likely to be successful in humans.

### (viii) What is the host response to SARS-CoV-2 and pathology of COVID-19?

After an initial push to look for therapeutic and vaccine options to help treat and prevent COVID-19, it will be important to better understand the host response to infection and the pathology of disease. A prerequisite step will of course be the development of appropriate animal models. Better understanding of how SARS-CoV-2 causes pathology and the way in which the host responds may help direct further therapeutic avenues. Understanding how comorbidities such as diabetes impact the host response to infection will also be important to better understand COVID-19.

### (ix) Why does SARS-CoV-2 appear to spread more rapidly than SARS-CoV or MERS-CoV in the human population?

Compared to the two other highly pathogenic coronaviruses that have emerged in the 21st century, SARS-CoV-2 appears to spread very well in the human population. Why this is the case will be interesting to understand. SARS-CoV and SARS-CoV-2 appear to use the same cell receptor of ACE2, suggesting a similar tropism, yet the novel coronavirus appears to spread much more efficiently simply based on the number of cases and the speed at which they have emerged. Whether proteolytic cleavage sites, such as a furin site in the spike protein of SARS-CoV-2, influence this will be important to determine for this outbreak and for the next. There are many other coronaviruses that have been found in bats that have potential for spread in the human population (24–26). Developing an understanding of the difference in the dynamics of spread between SARS-CoV-2 and the other coronaviruses will provide insights to understand which viruses may pose the most threat for zoonotic transmission and mass spread in the human population, leading to the final question…

### (x) What will be the next coronavirus to enter the human population?

The past 18 years have seen the emergence of three novel coronaviruses that have caused significant morbidity and mortality in the human population. Continued surveillance of viruses in animal populations and understanding the factors that influence zoonotic events are essential for attempts to limit future outbreaks.

### Conclusion.

The year 2020 has started with a rapid, global epidemic of the virus SARS-CoV-2, causing the disease COVID-19. The virus appears to have transmitted to humans in a zoonotic event from bats. There are many questions to investigate regarding all aspects of SARS-CoV-2 virology and epidemiology. These questions range from how the virus emerged to how it spreads and how the disease manifests. But most pressingly as the global outbreak continues to grow, can we develop effective vaccine and therapeutic strategies to treat not only this epidemic but any future coronavirus spillover events?
